# Effect of Counter-Surface Texturing on the Tribological Behavior of Virgin and Glass-Filled Polytetrafluoroethylene During Sliding Against Grey Cast Iron

**DOI:** 10.3390/polym18212742

**Published:** 2026-11-04

**Authors:** Mohammad F. Wani, Ferdous Ahad Bhat, Umida Ziyamukhamedova, Mohammad Jebran Khan, Lutfillo Bakirov, Jasurbek Nafasov, Elbek Turgunaliev

**Affiliations:** 1 Department of Materials and Mechanical Engineering, Tashkent State Transport University, Tashkent Temiryulchilar St., Tashkent 100167, Uzbekistanz.umida1973@yandex.ru (U.Z.); nafasovz@mail.ru (J.N.); 2 Mechanical Engineering Department, Public Works, Roads and Buildings (PWD-R&B), Jammu and Kashmir 190006, India; 3 Mechanical Engineering Department, SSM College of Engineering, Parihaspora Pattan, Kashmir 193121, India; 4 Department of Mechanical Engineering, Islamic University of Science and Technology, Awantipra, Kashmir 192122, India; 5 Department of Mechanical Engineering, Andijan State Technical Institute, 56 Baburshokh Street, Andijan 170100, Uzbekistan; 6 Department of Mechanical Engineering, Kimyo International University in Tashkent, 156 Usmon Nosir Street, Tashkent 100121, Uzbekistan

**Keywords:** polytetrafluoroethylene (PTFE), polymers, Laser surface texturing (LST), glass fibre

## Abstract

In this research study, the effect of laser surface texturing on the tribological performance of a composite of polytetrafluoroethylene with 25 wt.% glass fibre against grey cast iron was studied. Dimples (50 µm diameter) were ablated on grey cast iron with dimple pitches of 100 µm and 150 µm. Friction and wear tests were conducted on a pin-on-disc tribometer under ambient conditions. The specific wear rate of both polytetrafluoroethylene and its composite generally decreased with increasing sliding distance for all grey cast iron surfaces, whether textured or un-textured. The coefficient of friction followed a similar trend for polytetrafluoroethylene but increased for 25 wt.% glass fibre/polytetrafluoroethylene on textured grey cast iron surfaces. Compared to un-textured grey cast iron, polytetrafluoroethylene exhibited a 14.2–16.7% reduction in coefficient of friction and specific wear rate when sliding against grey cast iron with a 150 µm dimple pitch. For 25 wt.% glass fibre/polytetrafluoroethylene, laser surface texturing increased friction but significantly reduced the specific wear rate by 66.6–73.2%. Increasing the dimple pitch from 100 µm to 150 µm further decreased the coefficient of friction and wear. Field Emission Scanning Electron Microscopy micrographs of textured grey cast iron surfaces post sliding against polytetrafluoroethylene revealed polymer debris deposition, which led to the formation of transfer films that contributed to friction and wear reduction. These findings highlight the potential of laser surface texturing in enhancing the tribological performance of polytetrafluoroethylene-based materials, particularly in optimizing wear resistance and friction control through surface texturing.

## 1. Introduction

In food/beverage and pharmaceutical processing and packaging, automotive painting, textile processing, and chemical and petrochemical processing, air must be contamination-free. As a result, increased demand for oil-free reciprocating air compressors in such applications is inevitable. Oil-free compressors consist of a grey cast iron (GCI) cylinder and polytetrafluoroethylene (PTFE)-based piston rings, which ensure sealing as well as lubrication at the contact interface [1,2]. PTFE is a self-lubricating material that demonstrates a low coefficient of friction, which, however, signifies a high wear rate. Many research investigations have focussed on improving the wear resistance of PTFE, usually by reinforcement with micro- and nano-fillers, and significant improvements in the wear resistance have been achieved [3,4,5,6,7,8,9,10,11,12]. Nonetheless, glass fibre- and carbon-filled PTFE micro-composites have been used over the years for self-lubricating piston ring applications and are available commercially [13,14]. However, striving for improvement in performance is a continuous endeavour of scientists and researchers.

Laser surface texturing (LST) is one of the most useful methodologies for improving tribological properties, viz. the friction, wear and lubricating characteristics of all tribo-systems (bearings, shafts, piston rings, etc.). The most common and earliest application is honing, which is commonly seen in commercially available cylinder liners [15,16]. Various techniques have been developed and employed by researchers to obtain textured surfaces, viz. vibrorolling, reactive ion etching, abrasive jet machining, lithography and anisotropic etching, laser surface texturing (LST), etc., to improve the tribological performance [17,18,19,20,21,22,23,24,25,26,27,28,29]. However, LST at the micro- and nanoscale has proved to be an efficient and promising method compared to the others because it allows for excellent control of the shape and size of patterns, has a shorter processing time and has no environmental concerns. Moreover, LST has a wide range of applicability for different classes of materials (metals, ceramics and polymers) and hence is a promising idea [29]. Therefore, LST has gained significant attention from tribologists in recent years, and numerous studies have focussed on improving the friction and wear properties of materials using LST [30,31,32,33,34,35,36,37,38]. Recently, tribological studies on bio-inspired textures on metals and ceramic surfaces have shown tremendous potential [39,40,41]. Therefore, for improving the tribological properties of PTFE and its composite, LST has been applied.

Although several studies have investigated the influence of laser surface texturing on friction and wear behaviour, most of these studies have primarily focused on metallic or ceramic tribo-pairs under lubricated conditions. Limited attention has been given to polymer–metal tribo-systems involving PTFE-based composites sliding against grey cast iron, particularly under dry sliding conditions relevant to oil-free compressor applications. In addition, the combined influence of glass fibre reinforcement in PTFE and different texture densities on the tribological performance has not been sufficiently explored. Therefore, the present study aims to investigate the effect of laser surface texturing with different dimple pitches on the tribological behavior of virgin PTFE and glass fibre-reinforced PTFE when sliding against grey cast iron. The study further analyzes the corresponding wear mechanisms and transfer film formation using surface characterization techniques.

## 2. Experimental

### 2.1. Materials

**Figure 1:**
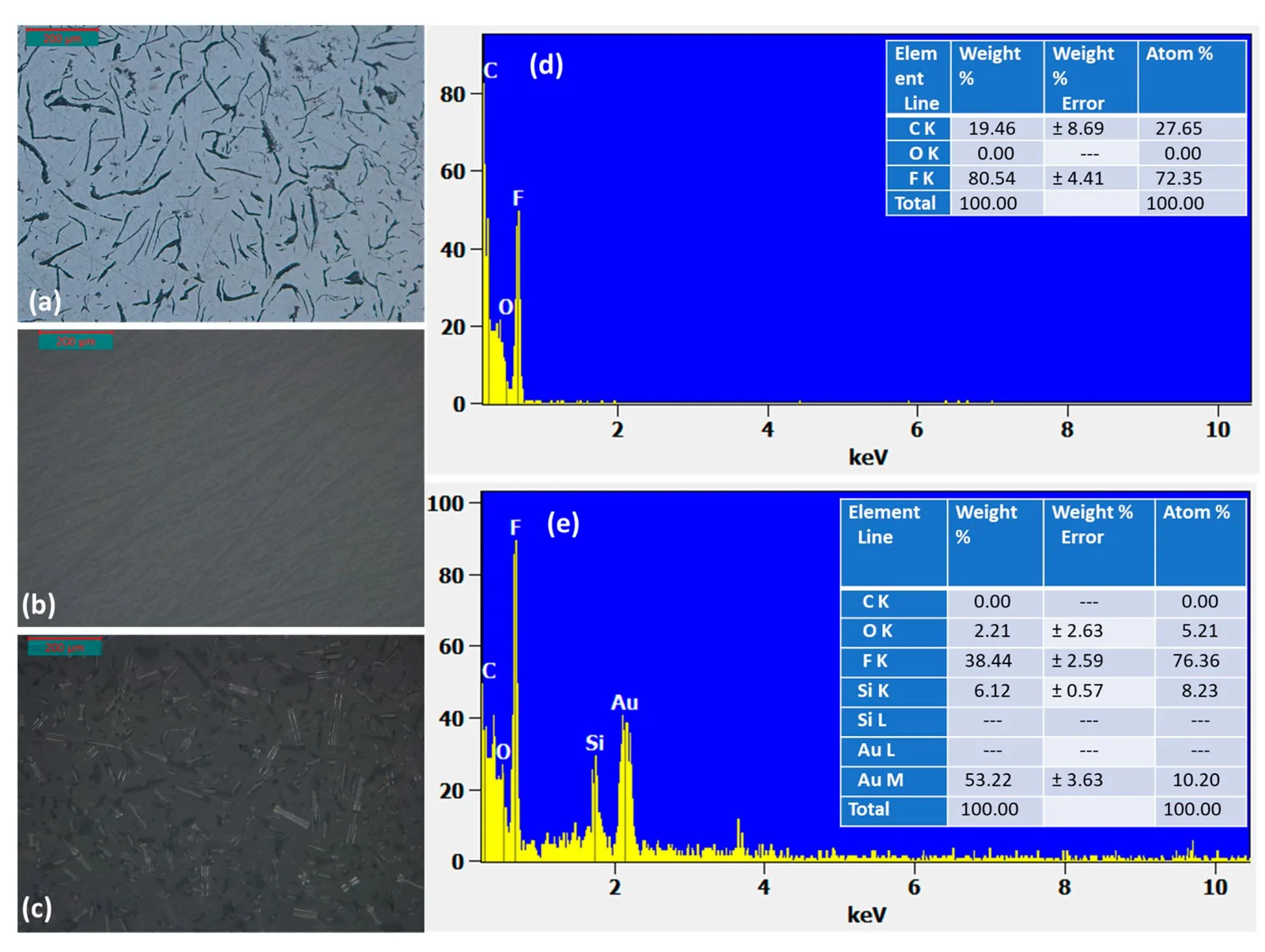
Optical micrographs of samples: (**a**) GCI; (**b**) virgin PTFE; (**c**) 25 wt.% glass fibre/PTFE. (**d**) and (**e**) EDS analysis of virgin and glass fibre/PTFE respectively.

**Figure 2:**
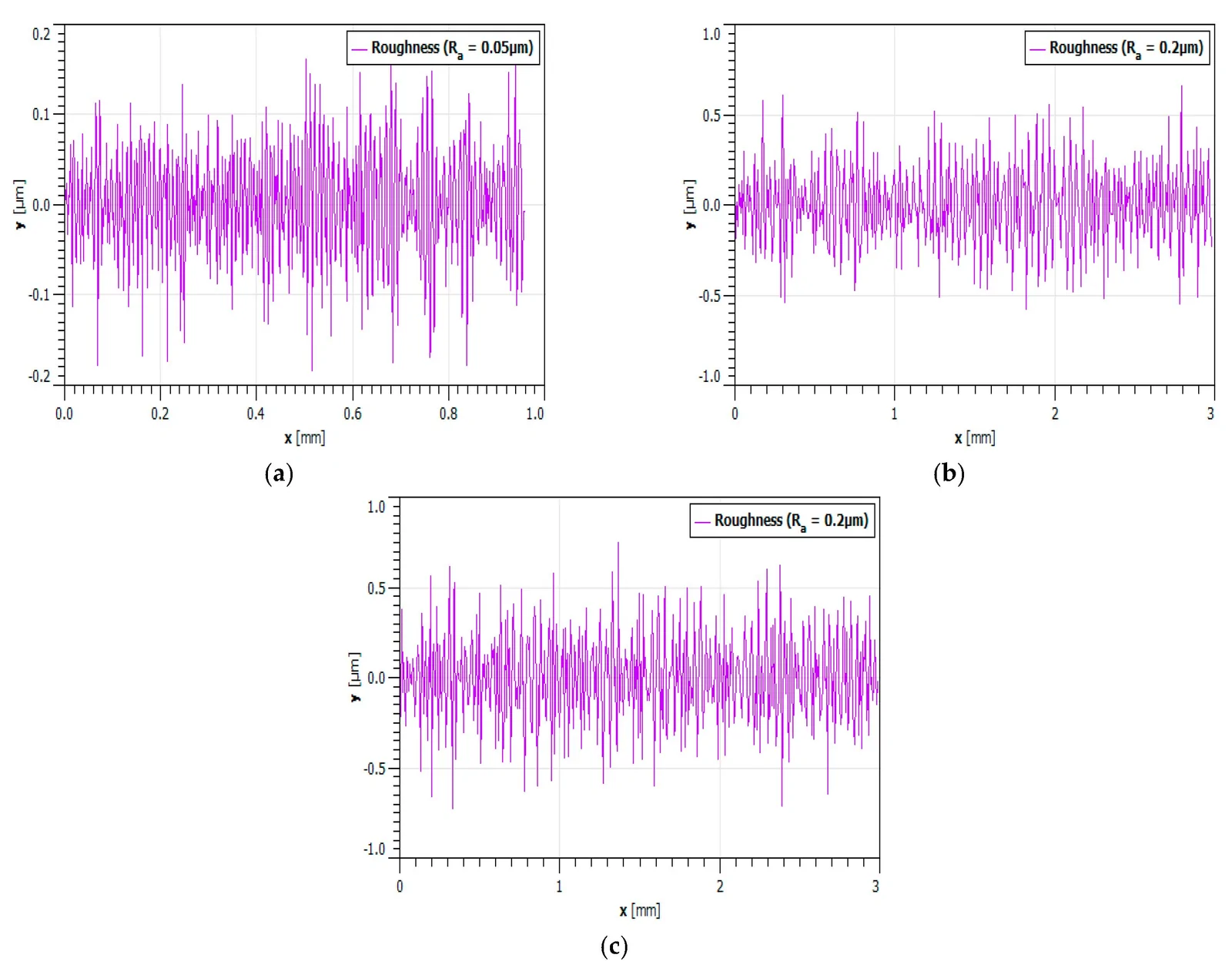
Surface roughness profiles of polished samples: (**a**) GCI; (**b**) virgin PTFE; (**c**) 25 wt.% glass fibre/PTFE.

The materials were procured from reliable sources in India. GCI was procured in the form of a cylindrical rod of 30 mm diameter, and disc samples of 10 mm height were cut from it. PTFE and its composites were procured from a reliable commercial vendor through the IndiaMART marketplace in India in the form of cylindrical rods of 8 mm diameter, which were further cut to pins of 10 mm in height and tapered to obtain a diameter of 3.5 mm at the testing end. The disc and pin samples were then polished using SiC papers and polishing cloths to obtain a fine surface finish. The optical micrographs, EDS and surface roughness of the polished samples are shown in Figure 1 and Figure 2, respectively.

The GCI substrate used in this study consists primarily of iron as the base element, with carbon in the range of 2.5–4.0 wt.% and silicon between 1.0 and 3.0 wt.%. Other alloying elements include manganese (0.2–1.0 wt.%), sulphur (0.02–0.25 wt.%), and phosphorus (0.02–1.0 wt.%). Trace amounts of nickel, chromium, and copper are also present. The microstructure of GCI is typically composed of graphite flakes embedded in a metallic matrix, consisting mainly of pearlite (ferrite–cementite lamellae) and ferrite. In addition, minor phases such as steadite eutectic and occasional cementite may also be present.

### 2.2. Laser Surface Texturing (LST)

**Figure 3:**
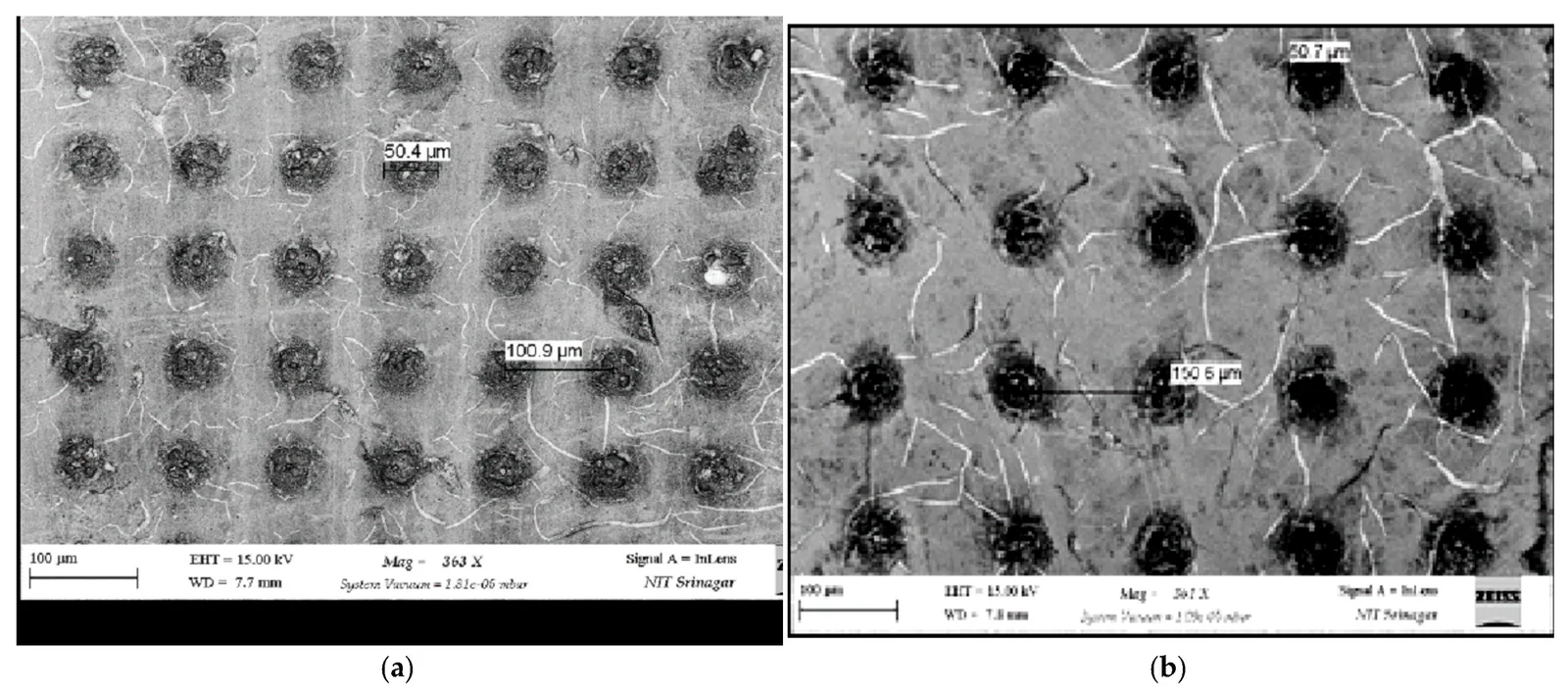
Textured surfaces of GCI: (**a**) 100 µm dimple pitch; (**b**) 150 µm dimple pitch.

**Figure 4:**
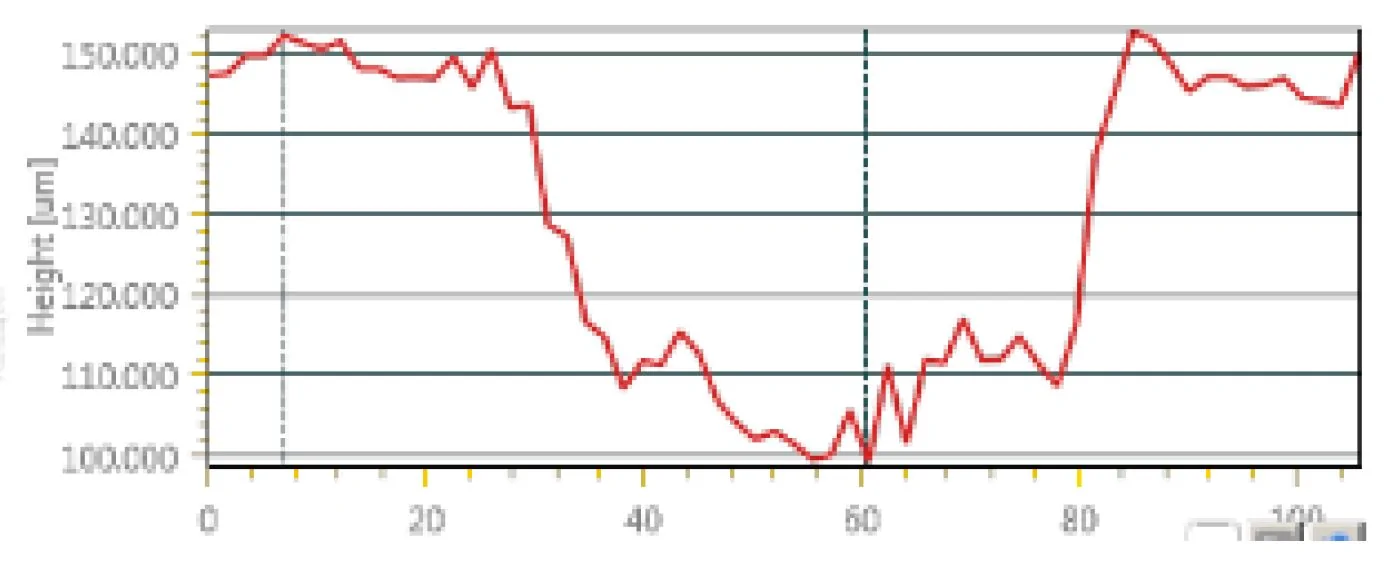
Three-dimensional profilometric trace of the dimple showing dimple depth.

LST was carried out on the polished GCI samples using laser radiation of wavelength 532 nm using a green laser. The laser radiation was run with a pulse width of 2.5 ns and a frequency of 10 kHz to yield hemispherical dimples. Dimples with a diameter of 50 µm and depth of 50 µm (average) were produced with varying dimple pitches of 100 µm and 150 µm. The FESEM images of the textured surfaces of GCI with dimple pitches of 100 µm and 150 µm are shown in Figure 3a and Figure 3b respectively. The dimple depth measured using an optical 3D profilometer is shown in Figure 4. Before tribo-testing, the samples were ultrasonically cleaned using ethanol for 10 min. No additional post-processing such as polishing or surface finishing was performed after laser texturing. The textured surfaces were used in the as-processed condition to preserve the original texture geometry produced by the laser. The samples were only ultrasonically cleaned with ethanol before tribological testing.

### 2.3. Experimental Design and Tribological Testing

In Table 1, tribological experiments were designed according to Taguchi’s L18 orthogonal array in order to obtain data in a controlled manner and were conducted on a pin-on-disc reciprocating sliding test rig under ambient conditions at room temperature (23 ± 1 °C). The tests were conducted at a constant normal load of 10 N, while the stroke, frequency and test duration were maintained at 5 mm, 10 Hz and 30 min respectively, corresponding to a sliding distance of 180 m. The sliding distance was varied in steps of 180 m up to 540 m. The control factors with their corresponding levels are shown in Table 1. The wear volume loss of PTFE pin samples was calculated using the weight loss method using a digital weight balance with an accuracy of 0.1 mg. Further, the wear rate was calculated using Equation (1). The experimental results were analyzed using S/N ratio analysis and ANOVA (analysis of variance) techniques. Furthermore, the wear surfaces were analyzed using optical microscopy and FESEM.

(1) $$\mathrm{Wear}\;\mathrm{Rate}\;({\mathrm{mm}}^{3}/\text{N-m})=\frac{\mathrm{Volume}\;\mathrm{loss}({\mathrm{mm}}^{3})\;}{\mathrm{Load}(N)\times \mathrm{Sliding}\;\mathrm{Distance}(m)}$$

## 3. Results and Discussion

### 3.1. Analysis of Friction

**Figure 5:**
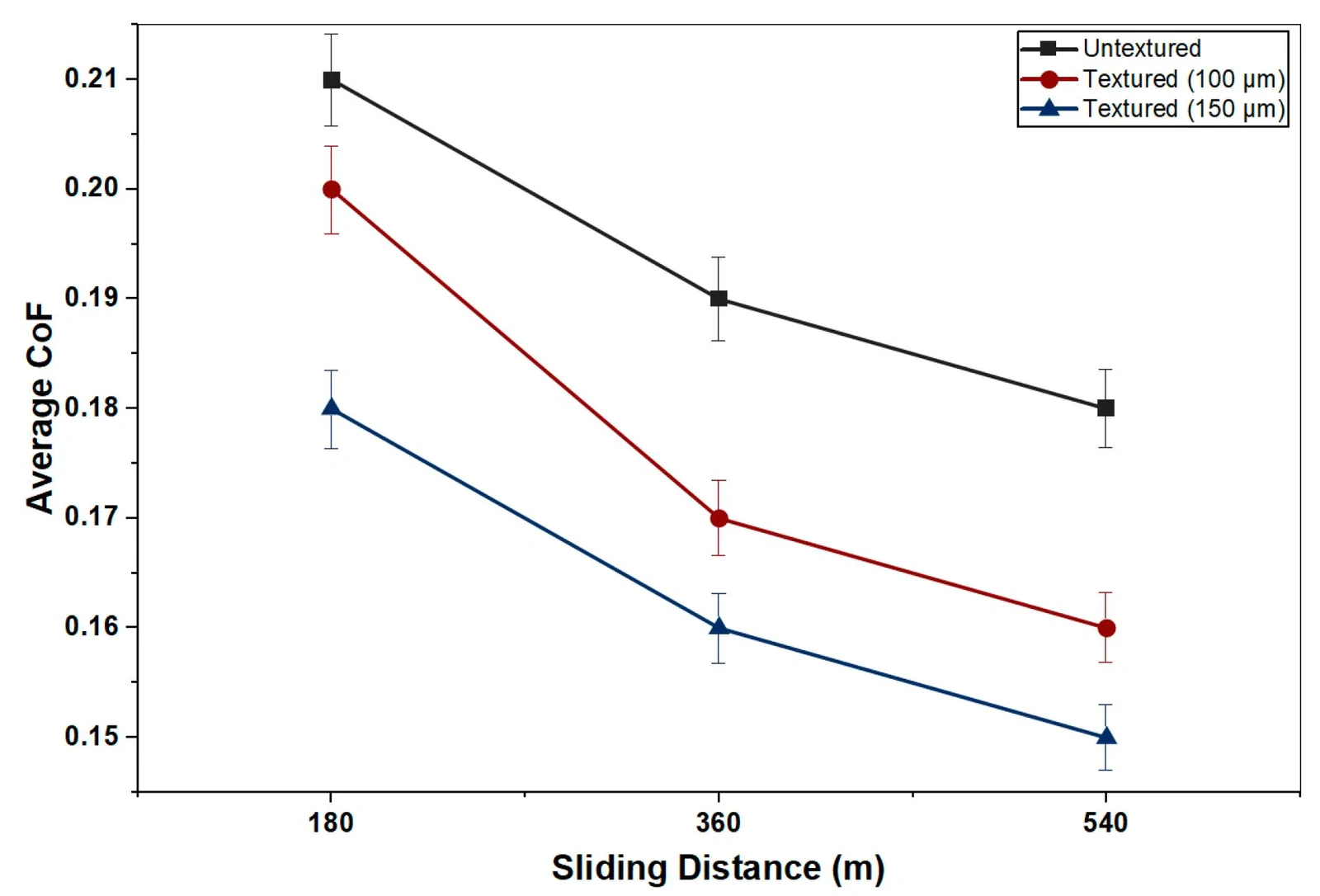
Average COF vs. sliding distance for virgin PTFE.

**Figure 6:**
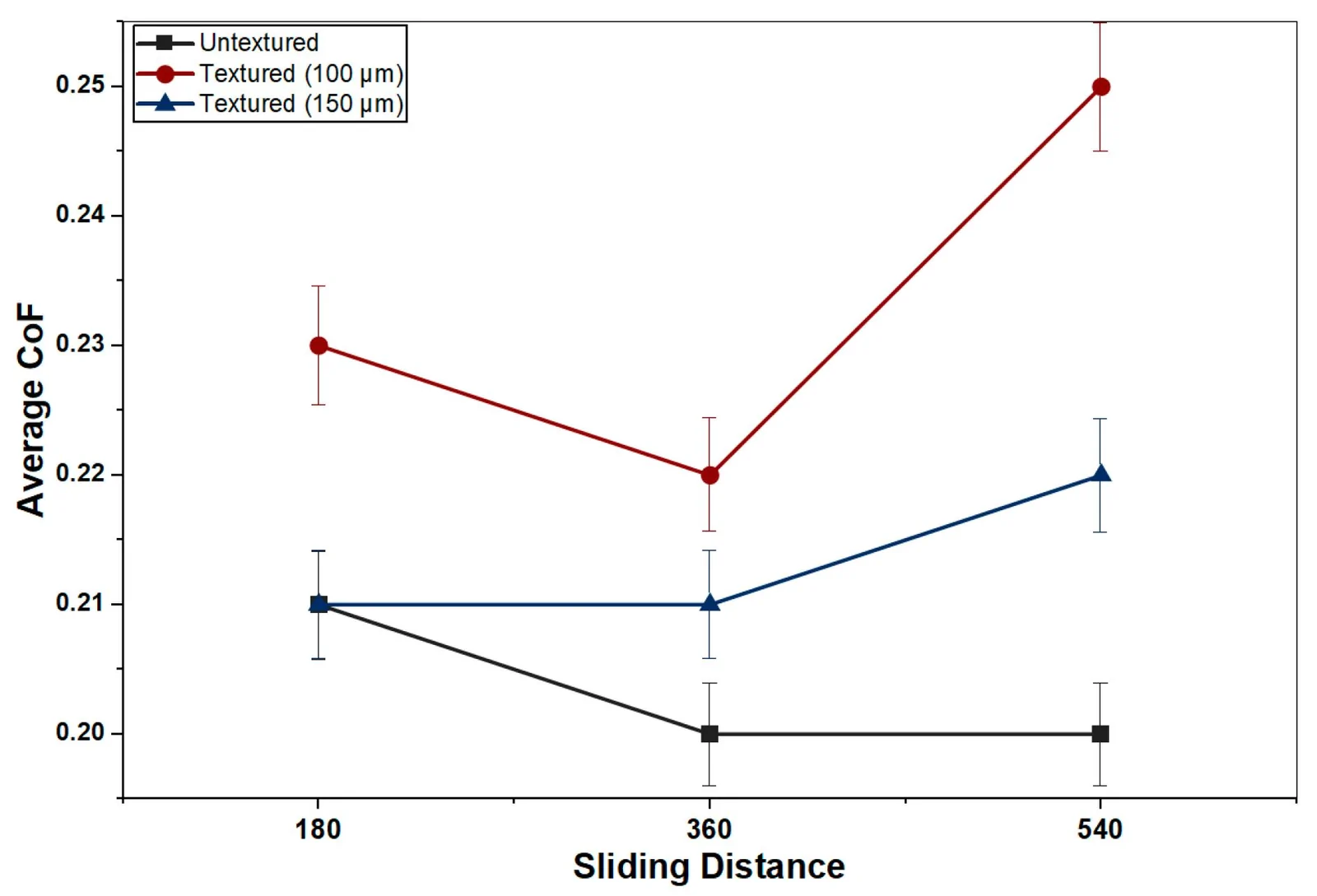
Average COF vs. sliding distance for 25 wt.% glass fibre-filled PTFE.

**Figure 7:**
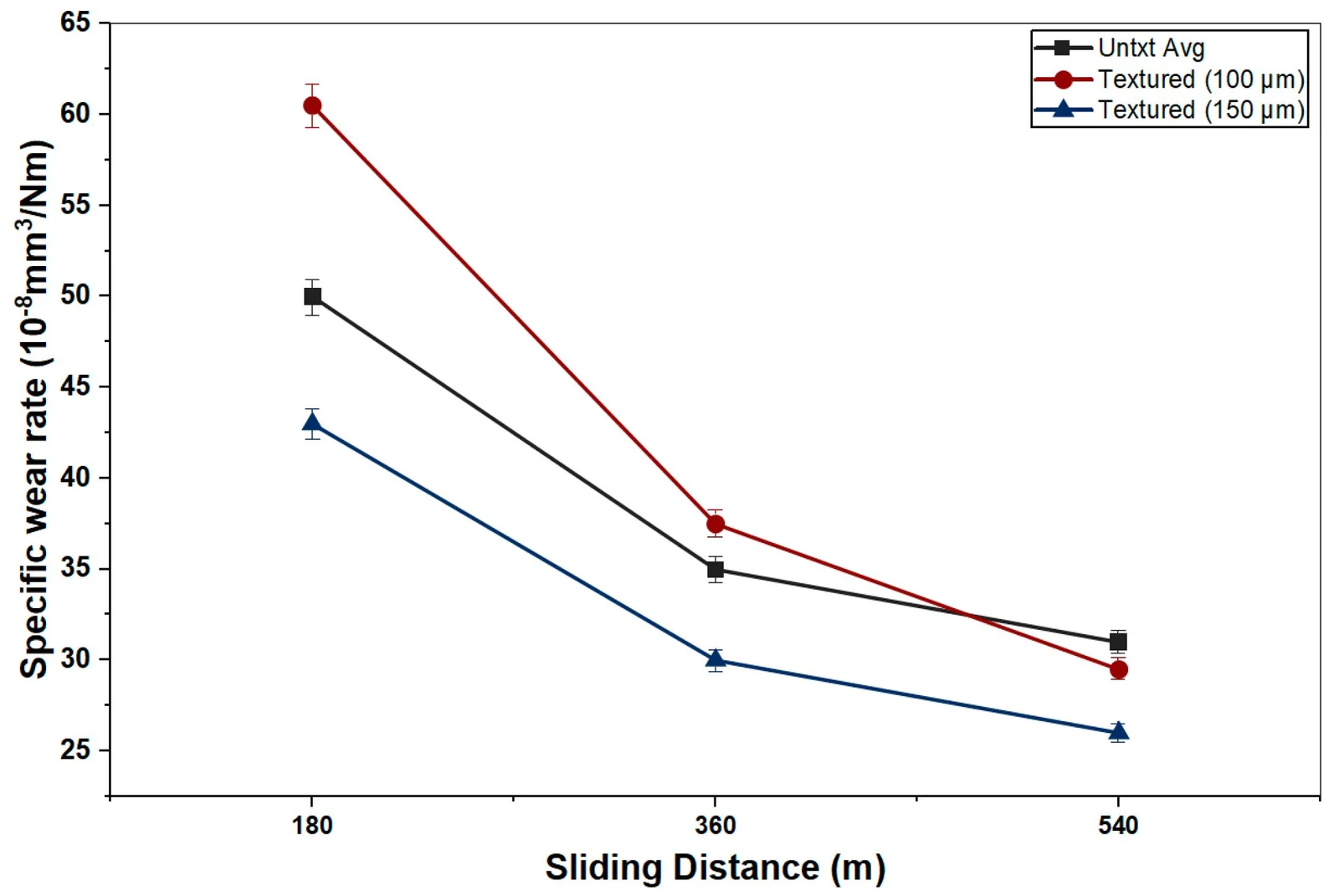
Specific wear rate vs. sliding distance for virgin PTFE.

**Figure 8:**
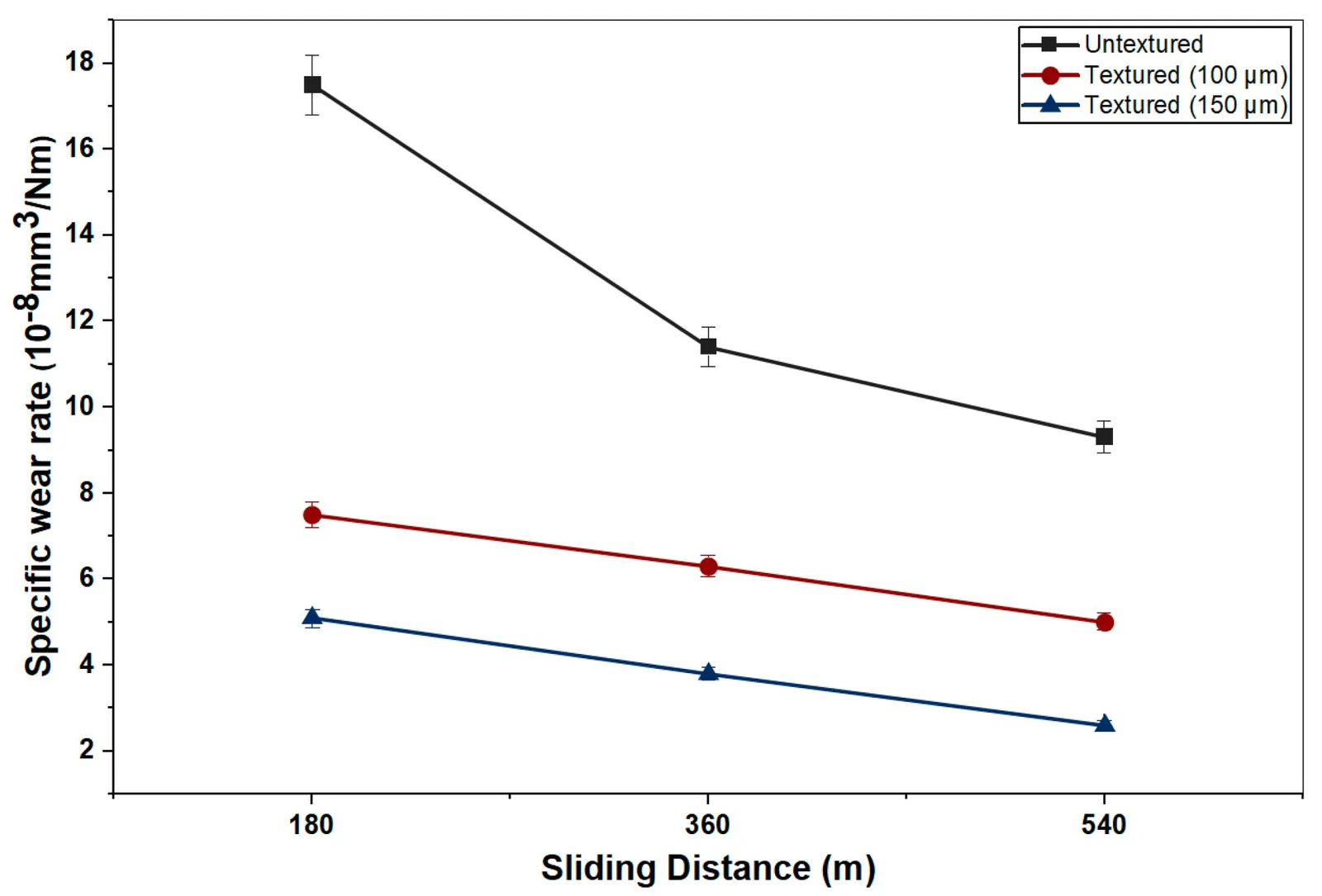
Specific wear rate vs. sliding distance for 25 wt.% glass fibre/PTFE.

The results of friction and wear tests are presented in Table 2 and Figure 5, Figure 6, Figure 7 and Figure 8. Figure 5 and Figure 6 show the variation in average friction coefficient versus sliding distance for virgin PTFE and 25 wt.% glass fibre-filled PTFE respectively while sliding against un-textured and textured grey cast iron surfaces. From Table 2 and Figure 5, it is evident that the average friction coefficient decreases with an increase in sliding distance for virgin PTFE, irrespective of the surface morphology of the counter surface. Moreover, it is observed that for virgin PTFE, the average friction coefficient is lowest against textured GCI with a 150 µm dimple pitch, irrespective of the sliding distance, followed by textured GCI with a 100 µm dimple pitch and then un-textured GCI. The friction coefficient of virgin PTFE decreased from 0.18 to 0.15 when sliding against textured GCI with a 150 µm dimple pitch compared to sliding against un-textured GCI at a 540 m sliding distance. However, the results are quite different for 25 wt.% glass fibre-filled PTFE when sliding against un-textured and textured grey cast iron surfaces. From Table 2 and Figure 6, it is clear that for 25 wt.% glass fibre-filled PTFE, the average friction coefficient is lowest against un-textured GCI, followed by textured GCI with a 150 µm dimple pitch and textured GCI with a 100 µm dimple pitch. From Table 2 and Figure 6, it is evident that when sliding against un-textured GCI, the friction coefficient of 25 wt.% glass fibre-filled PTFE decreases from 0.21 to 0.2 with an increase in sliding distance from 180 m to 360 m and then remains constant at 0.2 up to a sliding distance of 540 m. Moreover, while sliding against textured GCI (100 µm dimple pitch), the friction coefficient of 25 wt.% glass fibre-filled PTFE decreased from 0.23 to 0.22 with an increase in sliding distance from 180 m to 360 m and then increased to 0.25 up to a sliding distance of 540 m. Further, while sliding against textured GCI (150 µm dimple pitch), the friction coefficient of 25 wt.% glass fibre-filled PTFE remained constant at 0.21 with an increase in sliding distance from 180 m to 360 m and then increased to 0.22 when increasing the sliding distance to 540 m.

### 3.2. Analysis of Wear

Wear rates on the order of 10^−7^–10^−8^ mm^3^/Nm were obtained for virgin PTFE and 25 wt.% glass fibre-filled PTFE, whether sliding against textured on un-textured GCI counter surfaces, confirming that the wear rates remained in the mild wear regime.

Figure 7 and Figure 8 show the variation in the specific wear rate with sliding distance for virgin PTFE and 25 wt.% glass fibre-filled PTFE respectively, while sliding against un-textured and textured grey cast iron surfaces. From Table 2 and Figure 7, it is apparent that the specific wear rate decreases with an increase in sliding distance for virgin as well as 25 wt.% glass fibre-filled PTFE while sliding against GCI counter surfaces. A similar trend was found for average COF in the case of virgin PTFE. It is also observed from Figure 7 that the specific wear rate for virgin PTFE is lowest against the textured GCI counter surface with a 150 µm dimple pitch, irrespective of the sliding distance. Moreover, PTFE showed lower wear rates on initial sliding up to 360 m against un-textured GCI compared to sliding against the textured GCI counter surface with a 100 µm dimple pitch. However, with an increase in the sliding distance to 540 m, the textured GCI counter surface with 100 µm dimple pitch showed a lower wear rate for PTFE than un-textured GCI. In the case of 25 wt.% glass fibre-filled PTFE, the lowest wear rate was again observed against the textured GCI counter surface with a 150 µm dimple pitch, followed by the textured GCI counter surface with a 100 µm dimple pitch and un-textured GCI (Figure 8).

### 3.3. Analysis of Worn Surfaces

**Figure 9:**
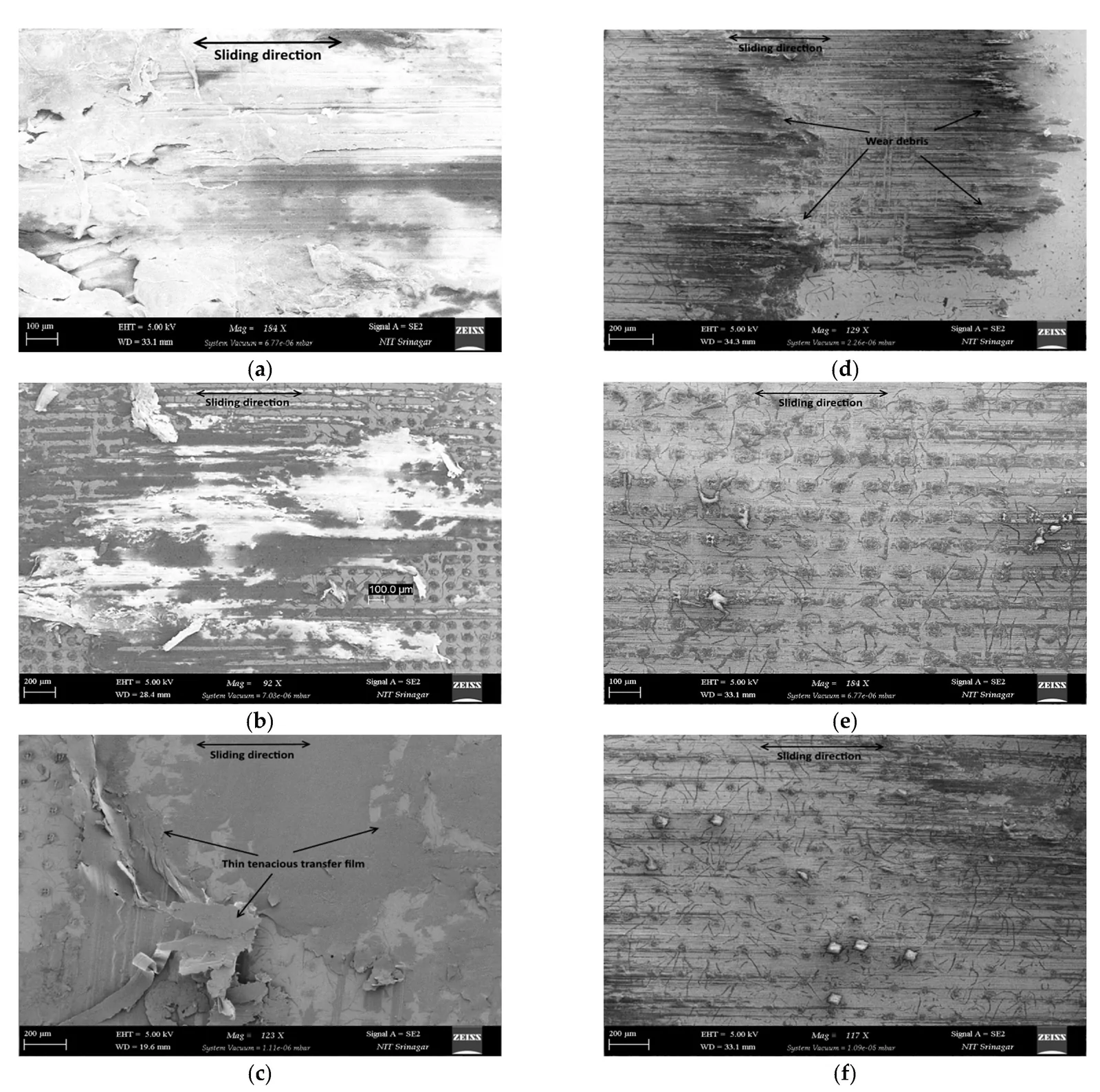
FESEM micrographs of the worn surfaces of the GCI counter surface after sliding a distance of 540 m: (**a**) un-textured GCI against virgin PTFE; (**b**) textured GCI with 100µm dimple pitch against virgin PTFE; (**c**) textured GCI with 150 µm dimple pitch against virgin PTFE; (**d**) un-textured GCI against 25 wt.% GF-filled PTFE; (**e**) textured GCI with 100µm dimple pitch against 25 wt.% GF-filled PTFE; (**f**) textured GCI with 150µm dimple pitch against 25 wt.% GF-filled PTFE.

**Figure 10:**
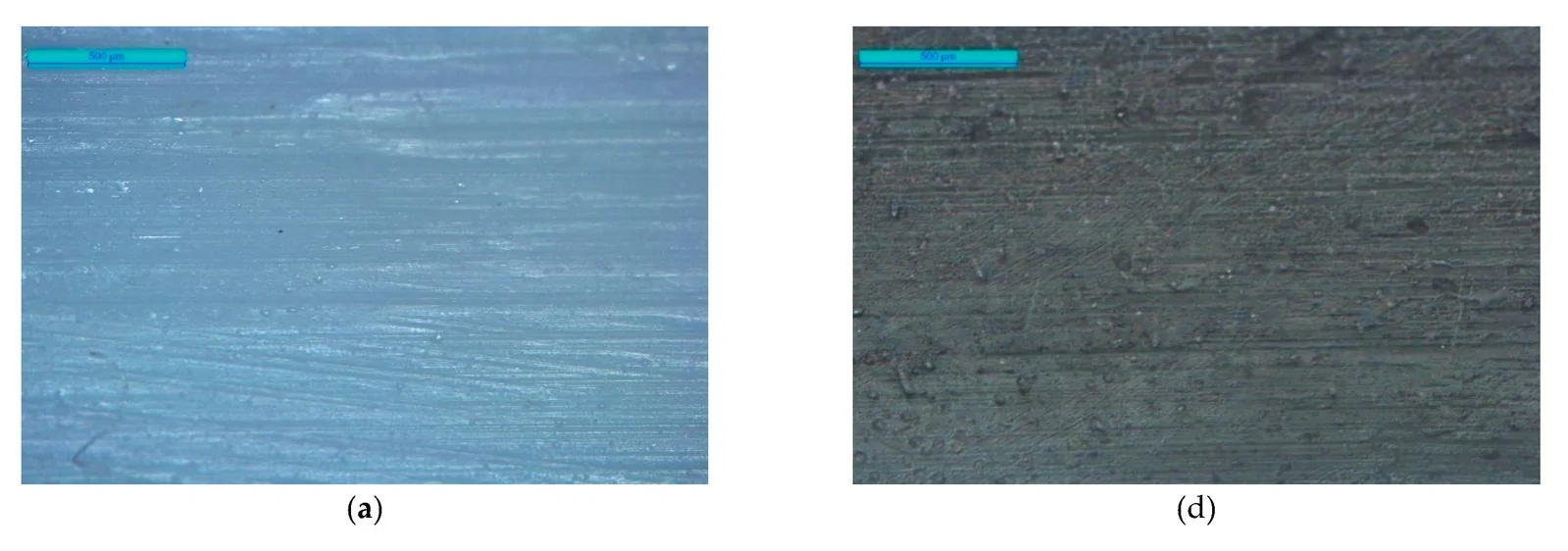

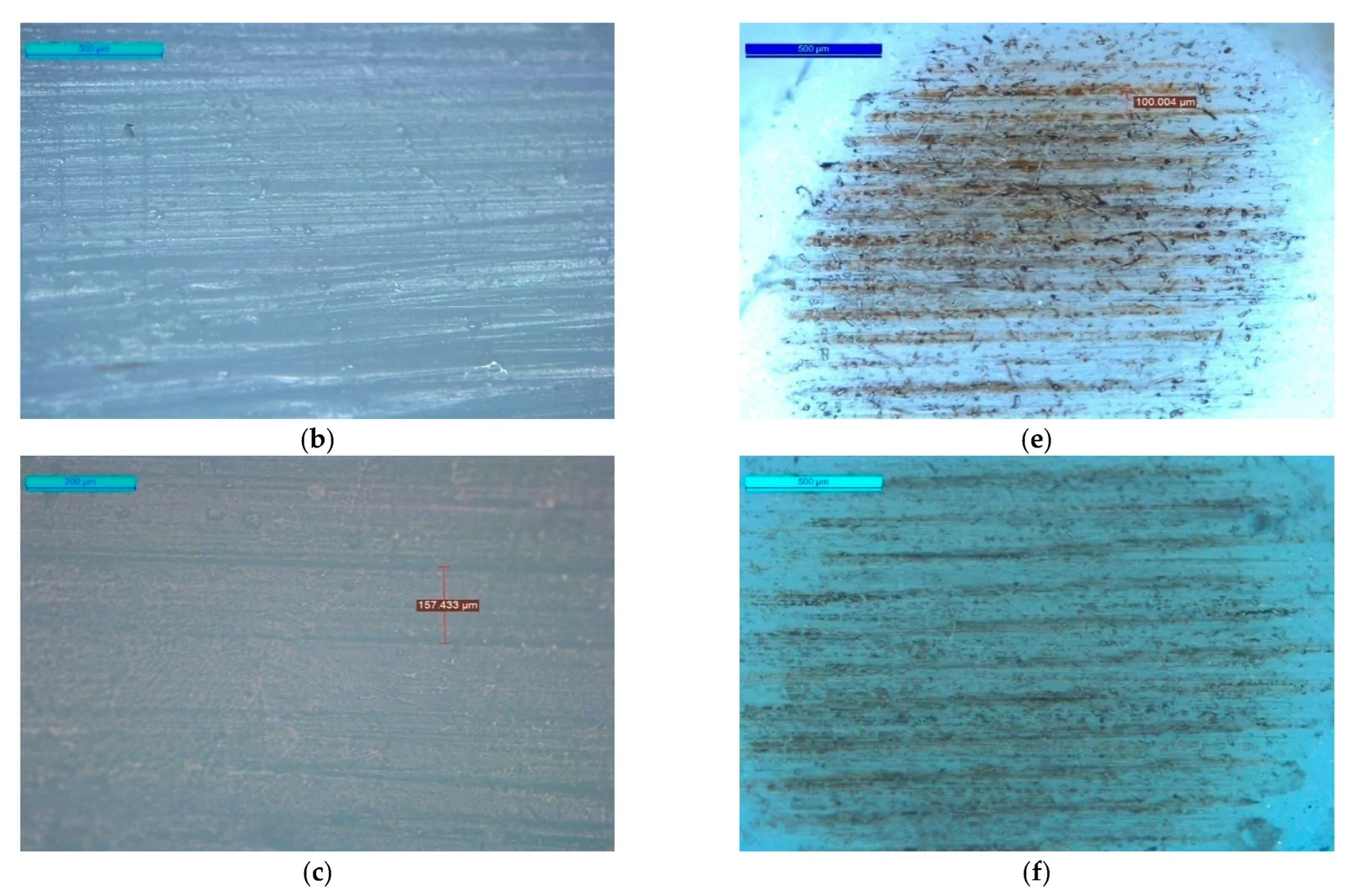
Optical micrographs of the worn surfaces of virgin PTFE and 25 wt.% GF-filled PTFE after sliding a distance of 540 m: (**a**) virgin PTFE against un-textured GCI; (**b**) virgin PTFE against textured GCI with 100µm dimple pitch; (**c**) virgin PTFE against textured GCI with 150 µm dimple pitch; (**d**) 25 wt.% GF-filled PTFE against un-textured GCI; (**e**) 25 wt.% GF-filled PTFE against textured GCI with 100µm dimple pitch; (**f**) 25 wt.% GF-filled PTFE against textured GCI with 150µm dimple pitch.

Figure 9a–c show the worn surfaces of the un-textured GCI counter surface, the textured GCI with 100 µm dimple pitch and the textured GCI with 150 µm dimple pitch after sliding for 540 m against virgin PTFE surfaces. It is evident from these figures that transfer of PTFE has taken place on the GCI surfaces in the case of both textured and un-textured surfaces. However, it is also evident from these figures that a thin and robust transfer film has formed on the GCI counter surface textured with 150 µm dimple pitch. This resulted in decreased friction and wear of the virgin PTFE against the counter surface textured with 150 µm dimple pitch. Although the experiments were conducted under controlled ambient conditions (23 ± 1 °C), it is important to note that in practical applications such as oil-free compressors, frictional heating may occur during operation. Temperature rises can influence the mechanical properties of PTFE and the stability of the transfer film formed on the counter surface. At elevated temperatures, PTFE tends to soften, which may promote transfer film formation but may also affect its stability under sliding conditions. Therefore, temperature effects could influence the friction and wear behaviour of PTFE-based materials and should be considered in future investigations under more realistic operating conditions. However, such a thin and robust film is not present on the un-textured GCI and textured GCI (100 µm dimple pitch) counter surfaces; certainly, high deposition of PTFE is observed on the surfaces. This indicates that the geometrical parameters of the textured surface deserve due consideration in LST as they affect the tribological performance. The worn surfaces of virgin PTFE after sliding against GCI counter surfaces also support this hypothesis, as it is evident from Figure 10a–c that slight abrasion is observed on the surface of virgin PTFE after sliding against the GCI counter surface textured with a 150 µm dimple pitch. However, high abrasion of the PTFE worn surface can be observed for virgin PTFE upon sliding against un-textured GCI and textured GCI (100 µm dimple pitch) counter surfaces. Figure 9d–f show the worn surfaces of the un-textured GCI counter surface, the textured GCI with a 100 µm dimple pitch and the textured GCI with a 150 µm dimple pitch after sliding for 540 m against 25 wt.% GF-filled PTFE surfaces. It is evident from Figure 9d–f that transfer of material has occurred onto the un-textured GCI counter surface as a result of sliding; however, the textured GCI surfaces, both with a 100µm dimple pitch and 150 µm dimple pitch, do not show such transfer of material; certainly, deformation of the surface textures is observed. On observing Figure 10d–f, it is apparent that abrasion accompanied with adhesive wear has occurred on the worn surface of 25 wt.% GF-filled PTFE upon sliding against un-textured GCI; however, minor abrasion is observed on the surface 25 wt.% GF-filled PTFE upon sliding against textured GCI, for both the 100 µm dimple pitch and 150 µm dimple pitch samples. Some of the GCI material adhered to the worn surfaces of 25 wt.% GF-filled PTFE upon sliding against textured GCI surfaces due to the interaction of the textured surfaces with hard glass fibres, which also led to increased friction between the 25 wt.% GF-filled PTFE and the textured GCI surfaces. However, wear remained low due to these interactions. The increased friction observed during sliding of glass fibre-reinforced PTFE against textured GCI surfaces may be attributed to the interaction between the hard glass fibres and the edges of the textured dimples. During sliding, exposed glass fibres can interact with the textured pits, leading to localized ploughing and mechanical interlocking effects. These interactions increase resistance to sliding and may also disturb the formation of a uniform PTFE transfer film, thereby contributing to higher friction coefficients.

### 3.4. Analysis of S/N Ratio (Taguchi Approach)

**Figure 11:**
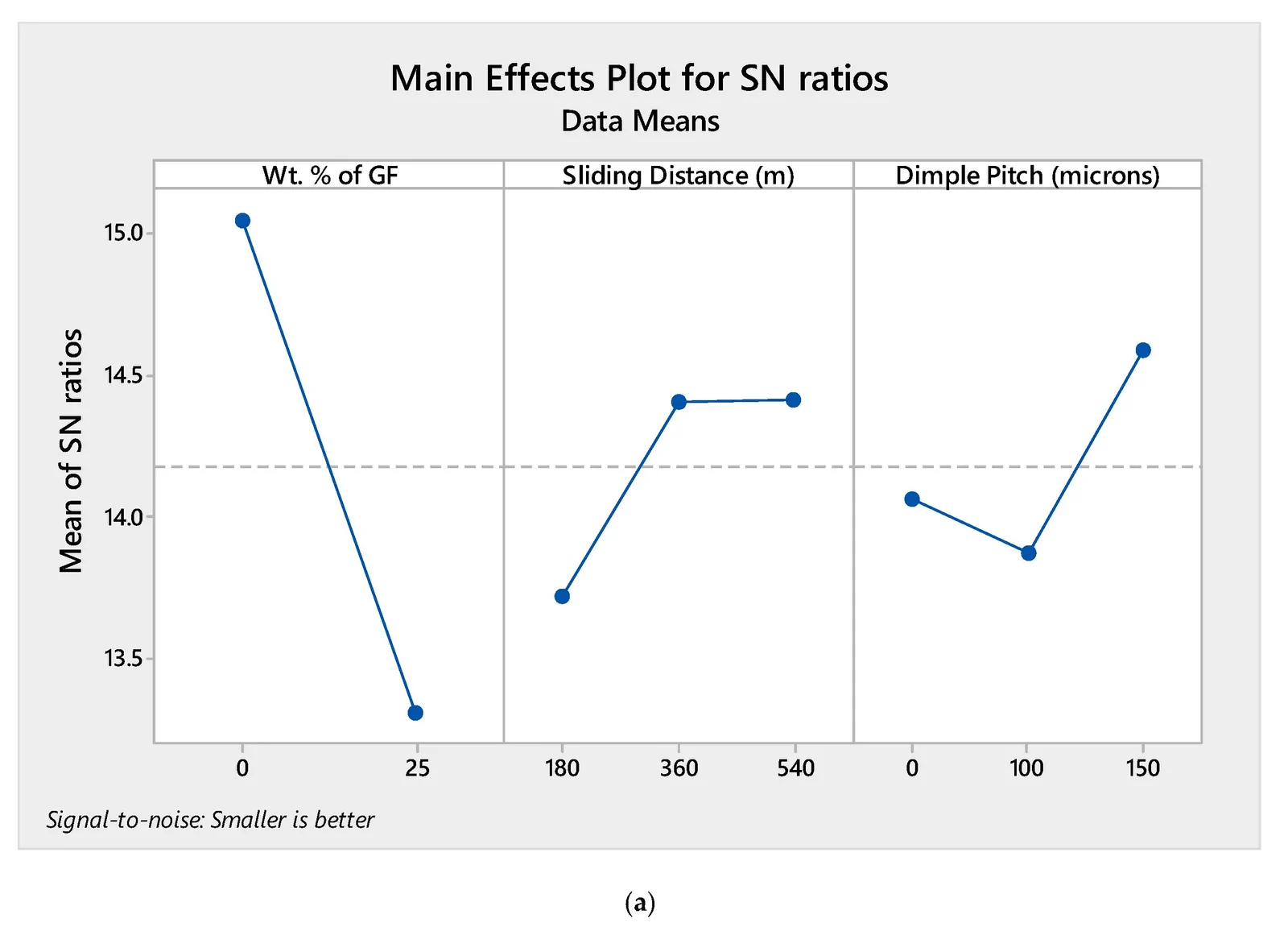

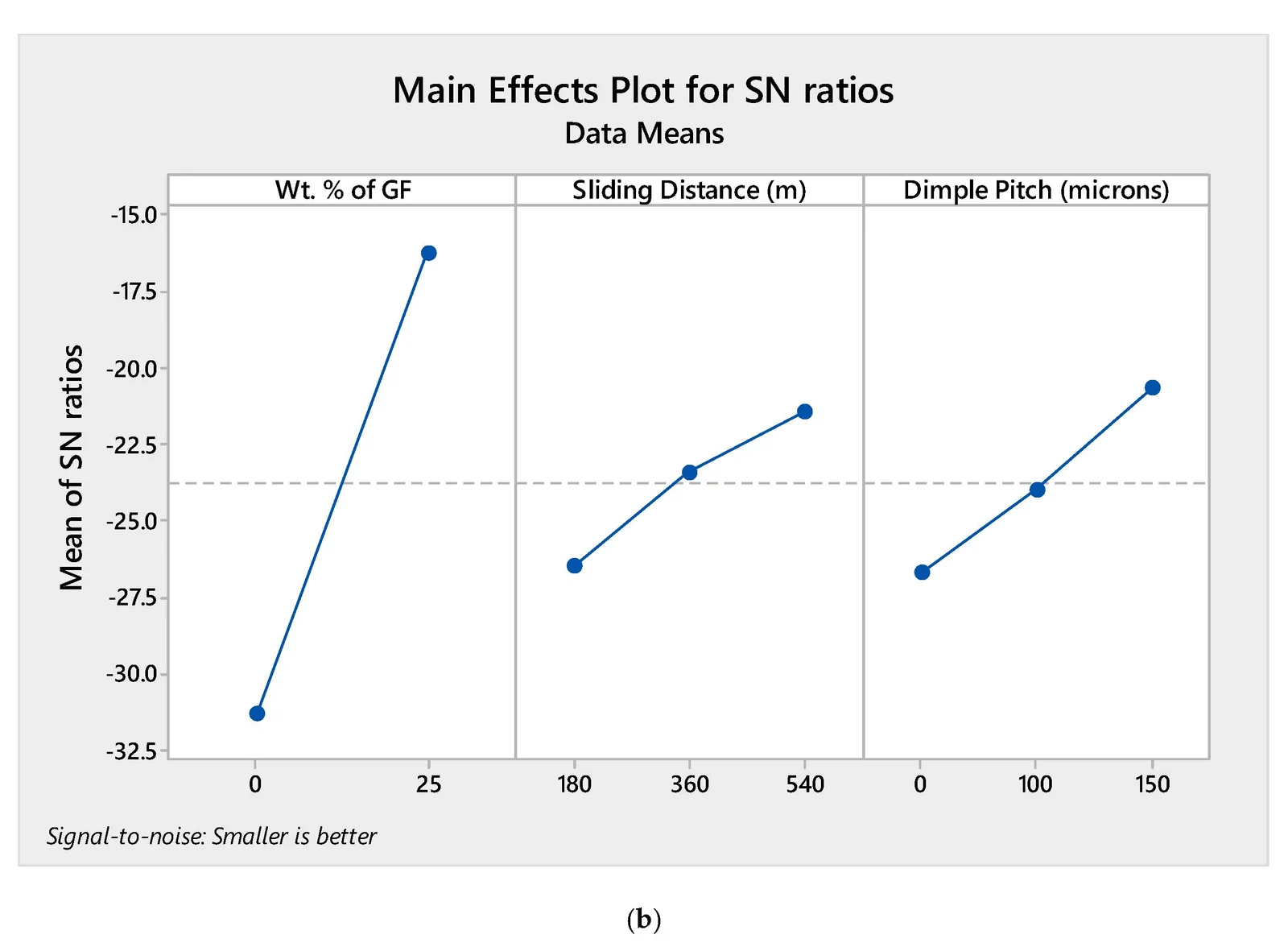
(**a**) Influence of control parameters on average S/N ratio for COF. (**b**) Influence of control parameters on average S/N ratio for wear rate.

The experimental results of friction and wear were analyzed statistically using Minitab software-1. The S/N ratio analysis was carried out to obtain the control factor settings for COF and wear rate. The S/N ratios for the obtained experimental values of COF and wear rate are shown in Table 2, and the S/N ratio response is shown in Table 3. Figure 11a,b demonstrate the effect of control factors (viz. wt.% age of GF, sliding distance and dimple pitch) on the average S/N ratio for COF and wear rate respectively. The highest values for the S/N ratios (bold values in Table 3) indicate the optimal factor settings for COF and wear rate. These bold values in Table 3 are indicated as the highest values for the S/N ratios in Figure 11a and Figure 11b respectively for COF and wear rate. From Table 3 and Figure 11a,b, it is evident that the optimal parameter settings for COF and wear rate are: wt.% age of GF: 0; sliding distance: 540 m; and dimple pitch: 150µm and wt.% age of GF: 25; sliding distance: 540 m; and dimple pitch: 150 µm respectively. Further, it is also evident from Table 3 that the wt.% age of GF is the most significant parameter affecting the COF and wear rate, followed by dimple pitch and sliding distance.

### 3.5. Analysis of Variance (ANOVA)

**Figure 12:**
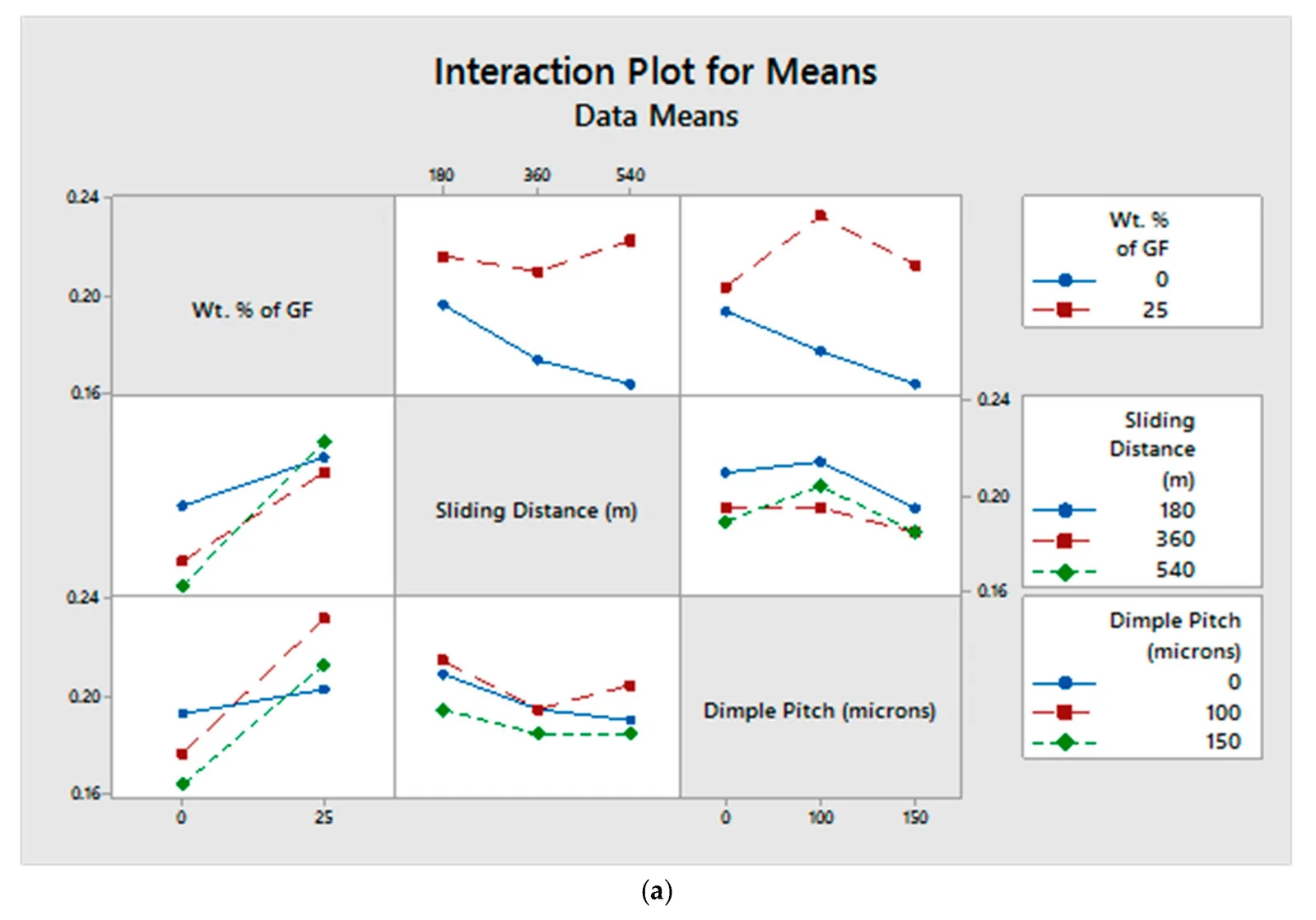

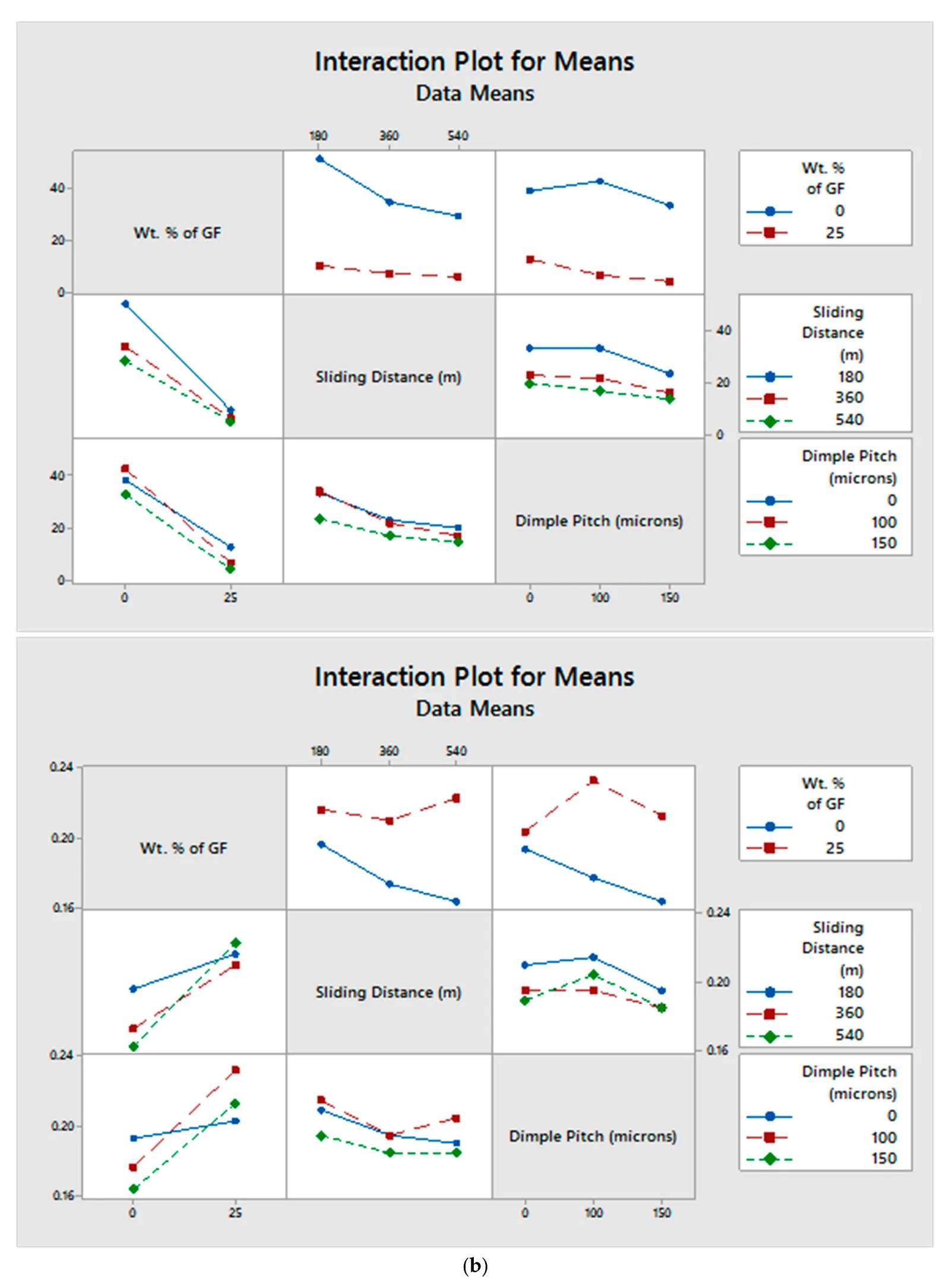
(**a**) Interaction of control parameters for average COF. (**b**) Interaction of control parameters for wear rate.

In order to reveal the significant control parameters, their interactions and their percentage contribution to COF and wear rate, the experimental results of friction coefficient and wear rate were subjected to analysis of variance. ANOVA was achieved at the 95% confidence level and 5% significance level using Minitab software, and the results are shown in Table 4 and Table 5 for average COF and wear rate respectively. It is evident from Table 4 that the wt.% age of GF is the most significant factor for COF, with a percentage contribution of 56.43%, followed by dimple pitch (6.11%) and sliding distance (5.84%). The interactions of wt.% age of GF*dimple pitch and wt.% age of GF*sliding distance are also significant, with percentage contributions of 15.04% and 9.2% respectively. The significance of these interactions can also be observed from the interaction plot of COF (Figure 12a). As suggested in [39], if the lines of an interaction plot interact with each other, then a significant interaction exists between the control parameters. From Table 5, it is evident that the wt.% age of GF is again the most significant parameter for wear rate, with a percentage contribution of 77.72%, followed by sliding distance (10.27%) and dimple pitch (3.02%). Moreover, the interactions of wt.% age of GF*dimple pitch and wt.% age of GF*sliding distance contribute to wear rate by 4.43% and 1.1% respectively. These nominal interactions are also confirmed by the interaction plot for wear rate (Figure 12b).

## 4. Conclusions

This study demonstrated that laser surface texturing is an efficient way to tailor the tribological characteristics of materials. This study showed that texturing the counter surface could improve the friction and wear characteristics of virgin PTFE, but at the same time showed that the geometrical parameters of the texture should be given important consideration in LST. For example, a dimple pitch of 150µm in the present case showed better results than a 100µm dimple pitch.

In the present study, the dimple diameter and depth were maintained constant at 50 µm to specifically investigate the influence of dimple pitch. However, the diameter and depth of the pits are also expected to influence the tribological performance, as deeper or larger dimples may enhance the debris trapping capacity and transfer film retention. Therefore, further optimization of the texture geometry by varying the pit diameter and depth may provide additional improvements in the friction and wear behaviour of PTFE-based materials.

This study also proved texturing the metallic counter surface could improve the tribo-characteristics of the soft polymer via robust transfer film formation; however, the same was not true for a polymer reinforced with hard fillers like glass fibres. The interaction of glass fibres with the micro-textured GCI surface upon sliding led to increased friction. The following conclusions can be drawn from this study:

The study concludes that the lowest average coefficient of friction (COF) and specific wear rate for virgin PTFE were observed against a textured grey cast iron (GCI) counter surface with a 150 µm dimple pitch, regardless of sliding distance. This was followed by textured GCI with a 100 µm dimple pitch and un-textured GCI in terms of average COF, whereas for specific wear rate, un-textured GCI performed better than textured GCI with a 100 µm dimple pitch. FESEM micrographs of the worn surfaces indicated the formation of transfer films on the textured GCI surfaces during sliding with virgin PTFE. Notably, a thin and robust transfer film was formed on the surface of textured GCI with a 150 µm dimple pitch, which contributed to reduced friction and wear. For 25 wt.% glass fibre-filled PTFE, the lowest specific wear rate was also observed against a textured GCI counter surface with a 150 µm dimple pitch, followed by textured GCI with a 100 µm dimple pitch and un-textured GCI. However, the lowest average COF for this material was recorded against un-textured GCI, followed by textured GCI with 150 µm and 100 µm dimple pitches. The signal-to-noise ratio (S/N) analysis identified the optimal factors for minimizing COF as virgin PTFE, a sliding distance of 540 m, and a dimple pitch of 150 µm, while the optimal settings for specific wear rate were 25 wt.% glass fibre/PTFE, a sliding distance of 540 m, and a dimple pitch of 150 µm. Analysis of variance (ANOVA) showed that the weight percentage of glass fibre was the most significant factor influencing both the COF and specific wear rate, contributing 56.43% and 77.72%, respectively. This indicates that under the present experimental conditions, the material composition (glass fibre content) plays a dominant role in governing the wear behaviour, while the influence of surface texture parameters, such as dimple pitch, is comparatively smaller. The contributions of sliding distance were 5.84% for COF and 10.27% for specific wear rate, while dimple pitch contributed 6.11% and 3.02%, respectively. Additionally, the interaction of glass fibre content and dimple pitch significantly influenced the COF and specific wear rate, with contributions of 15.04% and 4.43%, respectively. These findings highlight the crucial role of texturing, material composition, and operating conditions in optimizing the tribological performance of PTFE-based composites.

## Figures and Tables

**Table 1:** Control parameters and their levels.

Control Parameter	Units	Level		
		1	2	3
wt.% age of GF	%	0	25	-
Sliding distance	m	180	360	540
Dimple pitch	µm	0	100	150

**Table 2:** Results of friction and wear tests.

Exp. No.	wt.% of Glass Fibres	Sliding Distance (m)	Dimple Pitch (µm)	Average COF	Wear Rate (10^−8^ mm^3^/Nm)	S/N Ratio (COF)	S/N Ratio (Wear Rate)
1	0	180	0	0.21	50.0	13.5556	−33.9794
2	0	180	100	0.20	60.5	13.9794	−35.6351
3	0	180	150	0.18	42.7	14.8945	−32.6086
4	0	360	0	0.19	35.2	14.4249	−30.9309
5	0	360	100	0.17	37.7	15.3910	−31.5268
6	0	360	150	0.16	30.2	15.9176	−29.6001
7	0	540	0	0.18	31.1	14.8945	−29.8552
8	0	540	100	0.16	29.4	15.9176	−29.3669
9	0	540	150	0.15	25.9	16.4782	−28.2660
10	25	180	0	0.21	17.6	13.5556	−24.9103
11	25	180	100	0.23	7.5	12.7654	−17.5012
12	25	180	150	0.21	5.1	13.5556	−14.1514
13	25	360	0	0.20	11.4	13.9794	−21.1381
14	25	360	100	0.22	6.3	13.1515	−15.9868
15	25	360	150	0.21	3.8	13.5556	−11.5957
16	25	540	0	0.20	9.3	13.9794	−19.3697
17	25	540	100	0.25	5.0	12.0412	−13.9794
18	25	540	150	0.22	2.5	13.1515	−7.9588

**Table 3:** Response table for S/N ratios.

COF			
**Level**	**wt.% Age of GF**	**Sliding Distance (m)**	**Dimple Pitch (µm)**
1	15.05	13.72	14.06
2	13.30	14.40	13.87
3		14.41	14.59
Delta	1.75	0.69	0.72
Rank	1	3	2
**Wear Rate**			
1	−31.31	−26.46	−26.70
2	−16.29	−23.46	−24.00
3		−21.47	−20.70
Delta	15.02	5.00	6.00
Rank	1	3	2

**Table 4:** ANOVA for average COF.

Source	DF	Adj. SS	Adj. MS	F	*p*	% Age Contribution
wt.% age of GF	1	0.006806	0.006806	122.50	0.000	56.43
Sliding distance (m)	2	0.000811	0.000406	7.30	0.046	5.84
Dimple pitch (µm)	2	0.000844	0.000422	7.60	0.043	6.11
wt.% age of GF*Sliding distance (m)	2	0.001211	0.000606	10.90	0.024	9.2
wt.% age of GF*Dimple pitch (µm)	2	0.001911	0.000956	17.20	0.011	15.04
Sliding distance (m)*Dimple pitch (µm)	4	0.000156	0.000039	0.70	0.631	Pooled
Error	4	0.000222	0.000056			7.38
Total	17	0.011961				100

**Table 5:** ANOVA for wear rate.

Source	DF	Adj. SS	Adj. MS	F	*p*	%Age Contribution
wt.% of GF	1	4176.98	4176.98	345.59	0.000	77.72
Sliding Distance (m)	2	574.86	287.43	23.78	0.006	10.27
Dimple Pitch (µm)	2	186.06	93.03	7.70	0.043	3.02
wt.% of GF*Sliding Distance (m)	2	261.64	130.82	10.82	0.024	4.43
wt.% of GF*Dimple Pitch (µm)	2	83.05	41.53	3.44	0.135	1.1
Sliding Distance (m)*Dimple Pitch (µm)	4	27.57	6.89	0.57	0.700	Pooled
Error	4	48.35	12.09			3.46
Total	17	5358.50				100

## Data Availability

The original contributions presented in this study are included in the article. Further inquiries can be directed to the corresponding author.
